# STAR: A Privacy-Preserving, Energy-Efficient Edge AI Framework for Human Activity Recognition via Wi-Fi CSI in Mobile and Pervasive Computing Environments

**DOI:** 10.3390/s26123692

**Published:** 2026-06-10

**Authors:** Kexing Liu, Qiang Zhao, Rui Wang, Yuchu Lin, Jiahui Yu, Simon James Fong

**Affiliations:** 1 ZUMRI-DeepFuture Technology Joint Lab, The Zhuhai UM Science & Technology Research Institute (ZUMRI), Zhuhai 519000, China; 2 School of Communication and Information Engineering, Shanghai University, Shanghai 200444, China; 20820142@shu.edu.cn (Q.Z.); rwang@shu.edu.cn (R.W.); 3 School of Computer Science and Engineering, Macau University of Science and Technology, Macau SAR 999078, China; 3220001931@student.must.edu.mo; 4 Department of Computer and Information Science, Faculty of Science and Technology, University of Macau, Macau SAR 999078, China; yc47945@connect.um.edu.mo

**Keywords:** Wi-Fi CSI, human activity recognition, edge AI, privacy-preserving sensing, embedded edge devices, mobile IoT

## Abstract

Human activity recognition (HAR) using Wi-Fi channel state information (CSI) offers a privacy-preserving and contactless sensing modality suitable for smart homes, healthcare monitoring, and pervasive mobile Internet of Things (IoT) environments. However, existing CSI-based HAR approaches often suffer from computational inefficiency, high latency, and limited feasibility on resource-constrained embedded platforms. This work presents STAR (Sensing Technology for Activity Recognition), an edge AI-optimized framework that integrates lightweight temporal modeling, adaptive signal processing, and hardware-aware co-optimization to enable real-time, energy-efficient HAR on low-power embedded devices. STAR employs a streamlined three-layer Gated Recurrent Unit (GRU) architecture that reduces model parameters by 33% compared to conventional Long Short-Term Memory (LSTM) designs while maintaining strong temporal modeling capability. To enhance signal quality, STAR incorporates a multi-stage pre-processing pipeline consisting of median filtering, an eighth-order Butterworth low-pass filtering, and empirical mode decomposition (EMD) to denoise CSI amplitude measurements and extract stable spatial-temporal features. For on-device deployment, the system is implemented on a Rockchip RV1126 processor equipped with an embedded Neural Processing Unit (NPU) and interfaced with an ESP32-S3 CSI acquisition module. Experimental results demonstrate a mean recognition accuracy of 93.52% across seven activity classes and 99.11% for human-presence detection using a compact 97.6k-parameter model. INT8-quantized inference achieves a processing throughput of 33 MHz with only 8% CPU utilization, achieving a six-fold improvement in inference speed over CPU-based execution. With sub-second response latency and low power consumption, the system ensures real-time, privacy-preserving HAR, offering a practical, scalable solution for mobile and pervasive computing environments.

## 1. Introduction

In recent years, contactless human activity recognition (HAR) has emerged as a crucial technology for a wide range of applications, including smart homes [1,2], health monitoring [3], intelligent security [4], and smart city infrastructure [5,6]. These systems are increasingly adopted because they are non-intrusive, flexible, and suitable for both residential and commercial environments. In domestic settings, non-contact HAR systems are particularly valuable for monitoring elderly individuals and children [7,8], enabling continuous surveillance of daily activity patterns and providing early detection of critical anomalies such as falls, irregular sleep cycles, and prolonged inactivity. Such capabilities significantly reduce the caregiving workload while improving home safety. Furthermore, such systems support chronic disease management through passive monitoring of vital parameters such as respiratory rates and heart rate variability [9].

In commercial domains, HAR technologies facilitate customer demand analysis [10], optimize operational efficiency in office environments [11], and enhance public safety by enabling real-time detection of driver fatigue and distraction in transportation systems [12,13]. Likewise, they contribute to crowd density analysis, abnormal behavior detection, and infection risk assessment in public spaces [14]. Healthcare institutions, including nursing homes and hospitals, also leverage contactless monitoring to enhance caregiving efficiency, improve medical service quality, and reduce the incidence of unanticipated events through real-time vital sign detection [9,15].

Existing non-contact sensing techniques can be broadly categorized into vision-based, IoT-based, and radiolocation-based approaches. Each category offers different trade-offs in accuracy, privacy, environmental constraints, and deployment complexity. Vision-based methods, employing far-infrared thermography [16], near-infrared gesture recognition [17], and RGBD/visible-light cameras [18,19,20], offer effective activity tracking but are constrained by lighting conditions, line-of-sight requirements, and privacy concerns. IoT-based techniques infer human activities indirectly through environmental changes, utilizing pressure sensors, appliance usage logs [2], and acoustic monitoring [21]. Radiolocation-based methods, including millimeter-wave radar [12,22], ultra-wideband (UWB) systems [23,24], and RFID-based skeleton estimation [25], achieve motion capture by analyzing electromagnetic wave propagation and reflection characteristics. However, limitations such as multipath interference, material penetration issues, and elevated hardware costs impede their large-scale, low-cost deployment.

Wi-Fi channel state information (CSI) sensing has recently attracted considerable attention for indoor HAR due to its pervasive infrastructure, fine-grained channel characterization, and minimal privacy risks. Unlike traditional Received Signal Strength Indicator (RSSI)-based techniques, CSI captures millisecond-level fluctuations in wireless channels, enabling detailed motion characterization for diverse activities—from gross motor movements like walking and running [26] to subtle physiological phenomena such as breathing patterns [27] and finger movements [28]. Its capacity for concurrent multi-user tracking further enhances its suitability for multi-member smart home environments, making it a practical, scalable solution for real-time, non-invasive human monitoring.

The growing adoption of edge computing has accelerated the migration of HAR systems from centralized cloud-based infrastructures to decentralized, on-device implementations. Edge computing architectures offer key advantages in latency reduction, bandwidth savings, and privacy preservation—factors particularly critical for real-time surveillance and sensitive healthcare scenarios. However, conventional Wi-Fi sensing systems rely heavily on PCs or cloud servers for model training and inference, presenting challenges related to deployment complexity, network dependency, and data privacy. While emerging studies have investigated offline Wi-Fi sensing on edge devices, most have merely adapted existing PC-based models without optimizing for the computational, memory, and energy constraints inherent to embedded edge computing platforms.

**Figure 1:**
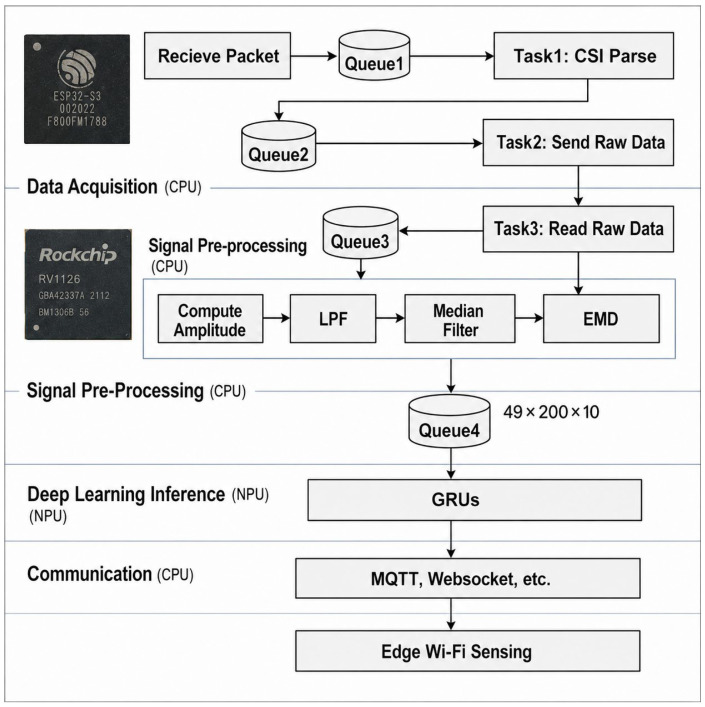
System overview of STAR, integrating digital signal processing techniques and deep learning models for edge Wi-Fi sensing.

To address these limitations, this paper proposes STAR (Sensing Technology for Activity Recognition), a Wi-Fi CSI sensing system specifically designed for low-power, resource-constrained embedded edge devices. As illustrated in Figure 1, STAR integrates a classical digital signal processing pipeline with a lightweight GRU-based recurrent neural network to model temporal CSI dynamics efficiently. Noise suppression and dimensionality reduction are achieved through median filtering, low-pass Butterworth filtering, and empirical mode decomposition (EMD), forming a compact and hardware-friendly pre-processing chain. For inference, STAR employs a streamlined Gated Recurrent Unit (GRU) architecture optimized for embedded Neural Processing Units (NPUs), enabling real-time temporal modeling with minimal computational overhead. System-level optimizations—including vectorized C implementations, ARM NEON acceleration, lock-free queues for latency-critical operations, and 8-bit integer quantization (INT8) quantized execution—further reduce latency and energy consumption. By offloading inference to the integrated NPU on the Rockchip RV1126, STAR achieves sub-second end-to-end latency while maintaining high recognition accuracy, demonstrating its suitability for practical deployment in mobile and pervasive computing environments.

In summary, this work makes the following contributions:

(1) We design a hardware-aligned CSI pre-processing pipeline tailored for embedded NPUs, balancing noise suppression with computational efficiency.

(2) We develop a lightweight GRU-based temporal model optimized for real-time inference under strict memory and latency constraints.

(3) We implement a complete end-to-end deployment workflow on the Rockchip RV1126, including quantization, NEON acceleration, and NPU offloading.

(4) We demonstrate sub-second, low-power HAR performance on real hardware, validating STAR as a practical embedded sensing solution.

The remainder of this article is organized as follows: Section 2 reviews related work in Wi-Fi-based HAR and embedded edge sensing systems. Section 3 details the design of the proposed system, including data acquisition, signal processing, and inference modules. Section 4 presents the experimental setup, covering data collection environments, model deployment configurations, and evaluation metrics. Section 5 concludes with a summary of findings and directions for future research.

## 2. Related Work

### 2.1. What Is the Best Way to Process CSI Data?

Deng et al. employed a Convolutional Neural Network (CNN)-based deep learning approach for human activity recognition (HAR) using Wi-Fi channel state information HAR via Wi-Fi CSI signals [29]. They proposed a lightweight deep learning model, WiLDAR, which processes CSI data through a CNN after dedicated pre-processing and feature extraction stages.

In a different approach, Hernandez et al. explored federated learning (FL) for processing Wi-Fi CSI signals [30]. Their framework, WiFederated, collaboratively trains machine learning models across multiple edge devices while safeguarding user privacy. Specifically, WiFederated enables each client to train locally on a subset of data and share only updated model parameters with a central server for aggregation, thereby reducing privacy risks associated with centralized raw data uploads.

### 2.2. Recent Advances in CSI-Based HAR

Hernandez and Bulut [31] investigated the feasibility of Wi-Fi sensing systems on edge devices and analyzed signal processing challenges and techniques. Through an extensive literature survey, they summarized commonly used signal processing methods and validated their effectiveness in HAR tasks of varying complexity. Their work provides a solid theoretical and practical foundation for the application of CSI in HAR.

Meanwhile, Deng et al. [29] further advanced the field by proposing WiLDAR, a lightweight model that combines stochastic convolutional kernels, depthwise separable convolutions, and residual structures. This architecture effectively reduces the network parameters and training time while achieving high accuracy, reinforcing the potential of CSI-based HAR on resource-constrained platforms.

The WiFederated framework [30,31] represents another significant contribution, integrating Wi-Fi sensing with federated learning to enable collaborative model training across multiple edge locations while preserving data privacy. By avoiding raw CSI data transfer, this approach enhances model robustness and adaptability across diverse environments.

Additionally, a study in [32] introduced a methodology for highly accurate through-the-wall wireless sensing using CSI and low-cost hardware. Using a Raspberry Pi 4B with an ALFA AWUS1900 adapter and Nexmon firmware for CSI extraction, the authors applied RNN- and LSTM-based deep learning models. Their systems achieved up to 97.5% classification accuracy even in challenging non-line-of-sight (nLoS) scenarios.

Furthermore, the authors of [33] presented an innovative method, applying the image processing techniques that are employed in Wi-Fi CSI-based HAR. By converting CSI data into RGB image representations and employing edge detection filters (Canny, Sobel, Prewitt, and LoG), they utilized 2D CNNs for classification of edge devices, improving both accuracy and training time. This work highlights how signal processing techniques can optimize model performance while reducing computational demands in edge deployments.

### 2.3. Comparison with LSTM and CNN Baselines

Prior CSI-based HAR studies have shown that GRU frequently matches or exceeds LSTM performance while requiring fewer parameters and offering lower latency, making it more suitable for embedded deployment [34,35]. CNN-based approaches, while effective for image-like CSI representations, typically incur higher memory and compute overhead and are less compatible with low-power NPUs. STAR, therefore, adopts a GRU-based architecture to balance accuracy with real-time constraints.

### 2.4. Motivation

In response to these developments, we propose a lightweight architecture specifically designed for edge computing environments. Our approach reduces the number of model parameters and computational complexity, incorporates an efficient network structure, and eliminates complex pre-processing steps by enabling end-to-end learning directly from raw CSI data. This design enhances inference speed and scalability while maintaining competitive accuracy across multiple deployment scenarios, offering a practical and advanced solution for edge-based Wi-Fi HAR.

## 3. Methodology

In this section, we present the specific structure of STAR. First, we introduce the data pre-processing of the proposed network. Then, the inference method and the classification module in STAR are subsequently analyzed.

### 3.1. Wi-Fi Signal Pre-Processing

Wi-Fi pre-processing of Wi-Fi CSI is a crucial step for improving signal quality and enhancing the robustness of downstream learning models. CSI amplitude measurements often contain noise from RF interference, hardware imperfections, and multipath fluctuations. The STAR pipeline applies a sequence of lightweight, hardware-friendly operations designed to suppress noise while preserving motion-related components.

#### 3.1.1. CSI Magnitude Extraction

Raw CSI consists of complex-valued subcarrier responses. To obtain a stable representation, STAR converts each complex coefficient into its magnitude *A*(*i*):

(1) $$\begin{array}{c} \left|A\left(i\right)\right|=\sqrt{{\left(h_{r}\left(i\right)\right)}^{2}+{\left(h_{i}\left(i\right)\right)}^{2}}, \end{array}$$

where $h_{r}\left(i\right)$ and $h_{i}\left(i\right)$ denote the real and imaginary components of the *i*th subcarrier, i.e., the in-phase and quadrature components, respectively. Magnitude information is more robust to phase noise and hardware-induced distortions, making it a widely adopted representation in CSI-based HAR.

#### 3.1.2. Median Filtering

A median filter is applied to suppress impulsive noise while preserving sharp transitions associated with human motion. For a sliding window of length *w* = 2*k* + 1, where *k* is the window radius, the CSI amplitude signal is a discrete time series *x*(*n*), and the median filtered output *y*(*n*) is as follows:

(2) $$\begin{array}{c} y\left(n\right)=median\left\{x\left(m\right)|m\in \left[n-k,n+k\right]\right\}, \end{array}$$

where the *median* {} denotes the median-taking operation. Boundary processing at the beginning and end of the signal is performed by reducing the window length, which is {0, *n + k*} if *n* < *k*. Median filtering is particularly effective for CSI because it removes burst-type interference without blurring temporal edges.

#### 3.1.3. Low-Pass Butterworth Filtering

To further suppress high-frequency fluctuations, STAR employs an 8th-order Butterworth low-pass filter. Butterworth filters provide a maximally flat passband, ensuring that low-frequency motion signatures are preserved while high-frequency noise is attenuated. The digital filter is implemented using the standard bilinear transform, as in Equation (3):

(3) $$\begin{array}{c} {\left|H\left(j\omega \right)\right|}^{2}=\frac{1}{1+{\left(\frac{\omega }{\omega c}\right)}^{16}}, \end{array}$$

where *ω* is the angular frequency (rad/s), and *ω_c_* is the cut-off angular frequency that determines the demarcation between the passband and stopband; 16 = 2 × *N*, where *N* = 8; the higher the filter order *N* is, the steeper the transition band. In the passband (*ω* < *ω_c_*), the amplitude response is as flat as possible; in the stopband (*ω* > *ω_c_*), the amplitude decays rapidly. The poles of a Butterworth filter lie on the unit circle of the complex plane and are uniformly distributed. For an 8th-order filter, the pole equation is as follows:

(4) $$\begin{array}{c} p_{k}=e^{j\left(\frac{\pi }{2}+\frac{\left(2k+1\right)\pi }{2N}\right)},k=0,1,\ldots ,7, \end{array}$$

where $p_{k}$ are the complex coordinates of the *k*th pole, *j* is an imaginary unit, and the poles are uniformly distributed on the unit circle to ensure that the amplitude response is maximally flat in the passband. The normalized low-pass filter design steps are divided into normalizing as follows: normalize the cut-off frequency, make *ω_c_* = 1, and design the prototype filter. Denormalization: mapping the prototype filter to the actual cut-off frequency via frequency transformation *ω_c_*. The bilinear transformation compensates for the nonlinearity of the frequency response by mapping the analogue frequency to the digital frequency through nonlinear frequency compression, which serves to convert the analogue transfer function *H*(*s*) to the digital transfer function *H*(*z*):

(5) $$\begin{array}{c} s=\frac{2}{T}\cdot \frac{z-1}{z+1}, \end{array}$$

*T*: sampling period (related to the CSI signal sampling rate); *z*: digital domain complex variable (*z* = *ejω*). The transfer function of the final digital filter is as follows:

(6) $$\begin{array}{c} H\left(z\right)=\frac{\sum_{k=0}^{8}b_{k}z^{-k}}{1+\sum_{k=1}^{8}a_{k}z^{-k}}. \end{array}$$

The numerator is calculated via the *scipy.signal.butter* function ($b_{k}$), and the denominator ($a_{k}$) coefficients are calculated.

Real-time computation is achieved by processing the CSI amplitude signal with difference equations, where each output sample *y*[*n*] is obtained via weighted summation of the current input *x*[*n*] and the historical inputs and outputs. The difference equation of the filter is given by the following:

(7) $$\begin{array}{c} y\left[n\right]=\sum_{k=0}^{8}b_{k}x\left[n-k\right]-\sum_{k=1}^{8}a_{k}y\left[n-k\right]. \end{array}$$

Each output sample *y*[*n*] is obtained via weighted summation of the current input *x*[*n*] and historical inputs and outputs. *x*[*n* − *k*]: past value of the input signal. *y*[*n* − *k*]: past value of the output signal.

The frequency response needs to be verified after design:

(8) $$\begin{array}{c} H\left(e^{j\omega }\right)=\frac{\sum_{k=0}^{8}b_{k}e^{-j\omega k}}{1+\sum_{k=1}^{8}a_{k}e^{-j\omega k}}, \end{array}$$

*ω*: digital corner frequency (range: 0 ≤ *ω* < *π*). Usually, a Butterworth filter passband without ripple is used. Stopband attenuation: 8th-order provides at least 48 dB/decade attenuation.

The 8th-order configuration offers a steep roll-off suitable for CSI amplitude signals, where high-frequency components typically correspond to noise rather than meaningful motion. The choice of an 8th-order Butterworth filter is motivated by the characteristics of CSI amplitude signals, which often contain high-frequency noise originating from RF interference, hardware imperfections, and rapid multipath fluctuations. Higher-order Butterworth filters provide a steeper roll-off while maintaining a maximally flat passband, allowing low-frequency motion-related components to be preserved while aggressively attenuating unwanted high-frequency artifacts. During system prototyping, lower-order filters (e.g., 2nd–4th order) were found to insufficiently suppress high-frequency fluctuations, whereas the 8th-order configuration offered a practical balance between noise reduction and computational cost. This design choice is consistent with prior CSI-based HAR pipelines that employ high-order filters to isolate human-motion signatures in the low-frequency band.

#### 3.1.4. Empirical Mode Decomposition (EMD)

EMD is applied to separate the CSI into intrinsic modal functions (IMFs), each representing oscillations at different frequency scales:

(9) $$\begin{array}{c} x\left(t\right)=\sum_{i=1}^{n}{\mathrm{IMF}}_{i}\left(t\right)+r_{n}\left(t\right), \end{array}$$

where *x*(*t*) is the original signal, IMF*i*(*t*) is the *i*th-order high-frequency to low-frequency Eigen mode function, and *r_n_*(*t*) is the residual signal. The process of signal reconstruction with its core equation is as follows:

(10) $$\begin{array}{c} x_{\mathrm{filtered}}\left(t\right)=\sum_{i=k}^{n}{\mathrm{IMF}}_{i}\left(t\right)+r_{n}\left(t\right). \end{array}$$

where *k* is the retained IMF starting order, and the filtering intensity is directly controlled by adjusting the value of *k* without complex parameter settings. In practice, high-frequency noise is usually located in the first few orders, and you can choose to remove the first few high-frequency IMF components; they can be removed as needed to smooth the signal. High-frequency IMFs, which predominantly contain noise, are removed before reconstruction. This adaptive decomposition is effective for CSI because it does not assume stationarity or linearity.

#### 3.1.5. Normalization and Subcarrier Selection

Finally, the pre-processed CSI data are normalized using Min–Max scaling:

(11) $$\begin{array}{c} x_{\mathrm{norm}}=norm\frac{x-x_{min}}{x_{max}-x_{min}}, \end{array}$$

where $x_{min}$ and $x_{max}$ are the minimum and maximum values of the signal, respectively. This operation removes the magnitude difference so that the data distribution satisfies $x_{\mathrm{norm}}$ ∈ [0, 1], which conforms to the distributional assumptions that most machine learning algorithms make about the input data.

STAR retains 49 subcarriers, which provide a compact yet informative representation. Adjacent subcarriers in 20 MHz Wi-Fi channels are highly correlated; thus, reducing from 52 to 49 subcarriers lowers computational cost with negligible loss of information—an important consideration for embedded NPU execution.

Although the ESP32-S3 provides 52 usable subcarriers under a 20 MHz channel, adjacent subcarriers exhibit strong spatial-frequency correlation due to the coherence bandwidth of indoor Wi-Fi channels. Retaining all 52 subcarriers, therefore, yields diminishing returns while increasing input dimensionality and memory footprint on embedded hardware. Selecting 49 subcarriers preserves the dominant spatial-frequency structure of the CSI magnitude while reducing the input size by approximately 6%, which directly benefits real-time inference on the RV1126 NPU. This choice reflects a practical trade-off between representational richness and computational efficiency and aligns with observations in prior CSI-based HAR studies where modest subcarrier reduction does not degrade recognition performance.

### 3.2. Inferences Method

**Figure 2:**
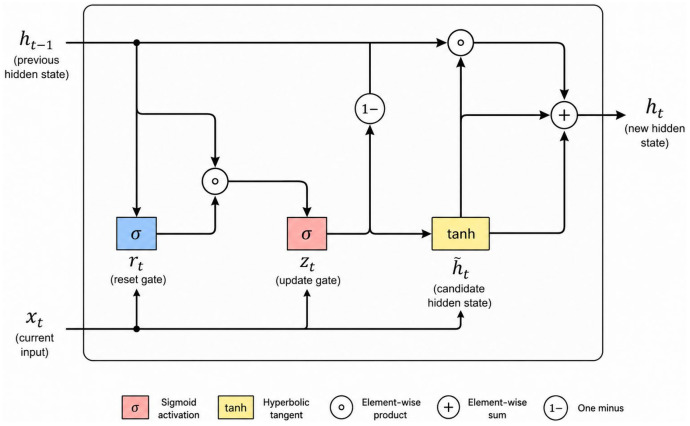
GRU cell architecture.

STAR employs a lightweight GRU network variant, proposed by Cho et al. [36,37,38,39], to model the temporal evolution of CSI magnitude sequences. As shown in Figure 2, GRU is a simplified recurrent architecture that uses two gates—an update gate and a reset gate to regulate information flow. Compared to LSTM, GRU requires fewer parameters while maintaining comparable modeling capacity, making it well suited for embedded edge devices.

At each time step *t*, the GRU computes the following:

(12) $$z_{t}=\sigma \left(W_{z}x_{t}+U_{z}h_{t-1}\right),\;r_{t}=\sigma \left(W_{r}x_{t}+U_{r}h_{t-1}\right)$$

(13) $${\tilde{h}}_{t}=tanh\left(W_{h}x_{t}+U_{h}\left(r_{t}⨀h_{t-1}\right)\right)$$

(14) $$h_{t}\left(1-z_{t}\right)⨀h_{t-1}+z_{t}⨀{\tilde{h}}_{t}$$

This structure enables efficient modeling of short- and medium-term dependencies in CSI sequences while avoiding the computational overhead of LSTM. Prior studies in CSI-based and RF-based HAR have shown that GRU often matches or exceeds LSTM performance with significantly lower latency and parameter count, further supporting its suitability for real-time edge deployment.

Recurrent neural architectures are widely used for modeling the temporal dynamics of Wi-Fi CSI signals, and among them, the Gated Recurrent Unit (GRU) provides an especially favorable balance between accuracy and computational efficiency. Prior work in CSI-based and RF-based human activity recognition has demonstrated that GRU frequently matches or exceeds the performance of LSTM while requiring substantially fewer parameters and offering lower latency, making it well suited for embedded and low-power platforms. For example, Zhang et al. report that GRU achieves higher accuracy than LSTM for CSI-based activity recognition with significantly reduced computational cost [34], and similar findings are observed in GRU-based HAR by Chen et al. [35]. These domain-specific results are consistent with large-scale evaluations of recurrent architectures showing that GRU provides competitive sequence-modeling capability with faster convergence and reduced memory footprint compared to LSTM [36,37,38,39]. Taken together, these studies provide strong empirical justification for adopting GRU as the core temporal modeling component in Sensing Technology for Activity Recognition (STAR), where real-time inference, low power consumption, and efficient deployment on embedded Neural Processing Units (NPUs) are primary design objectives.

The GRU has multiple advantages over traditional RNNs and LSTM. First, the simplified gating mechanism drastically reduces the number of parameters, and it reduces the computational complexity by approximately 33% without significantly affecting the model performance [38,39]. Empirical studies have shown that the GRU performs comparably to the LSTM in a wide range of sequence modeling tasks but tends to perform better in small datasets and tasks that require capturing short-term dependencies [40]. In addition, the GRU has shorter computational paths and smoother gradient flow, effectively mitigating the gradient vanishing problem [41].

Empirical studies have shown that GRUs perform well in a wide range of sequence modeling tasks. A large-scale evaluation of more than 10,000 RNN architectures by Jozefowicz et al. [38] revealed that the performance of GRUs in language modeling tasks is comparable to that of more complex models. In terms of computational efficiency, a study by Britz et al. [39] revealed that the GRU is, on average, approximately 25% faster than the LSTM in training time, and is only approximately 1–2% less accurate in neural machine translation tasks.

**Figure 3:**
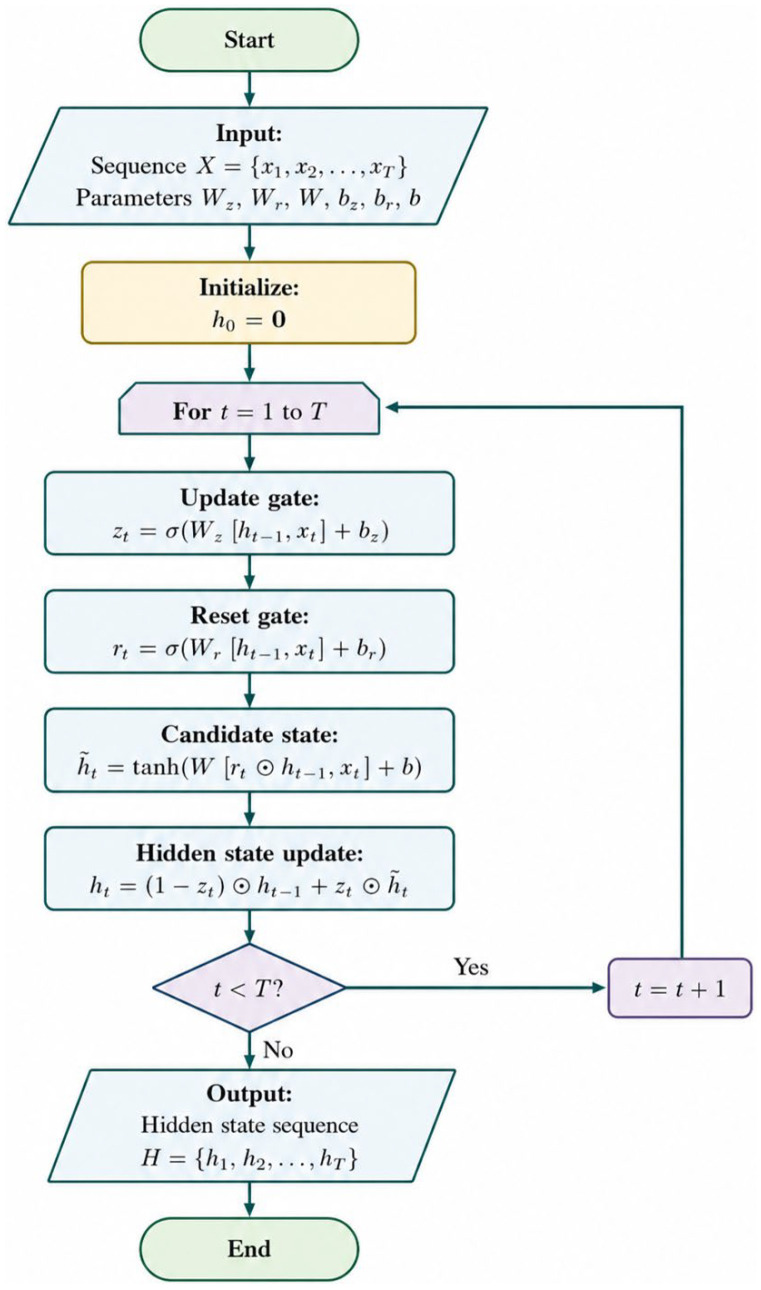
Flowchart of the GRU forward propagation algorithm.

The GRU model effectively solves the long-term dependency problem in traditional RNNs through its unique gating mechanism and achieves excellent performance in a variety of sequence modeling tasks. Its simplified structure not only reduces computational complexity but also alleviates the gradient vanishing problem, making it an efficient choice for processing sequence data. With the development of deep learning, the GRU has become an important basic model for time series data processing, providing strong support for many cutting-edge applications. Figure 3 shows the operation in flowchart.

Several design choices in STAR are guided by the constraints of embedded edge deployment and by empirical observations during system prototyping. An 8th-order Butterworth filter is employed to provide the steep roll-off necessary to suppress high-frequency noise in CSI amplitude signals while preserving low-frequency motion components. The selection of 49 subcarriers reflects a balance between representational richness and computational efficiency, as adjacent subcarriers in 20 MHz Wi-Fi channels are highly correlated, and retaining all 52 subcarriers yields minimal additional information while increasing latency. The GRU configuration is chosen to match the memory and throughput characteristics of the RV1126 NPU; GRU offers lower parameter count and faster convergence than LSTM while maintaining competitive accuracy, as demonstrated in prior CSI-based HAR studies [34,35]. These considerations collectively ensure that STAR achieves real-time performance under the resource constraints of embedded hardware. 

## 4. Experimentation

### 4.1. Data Acquisition

#### 4.1.1. Acquisition Device

**Figure 4:**
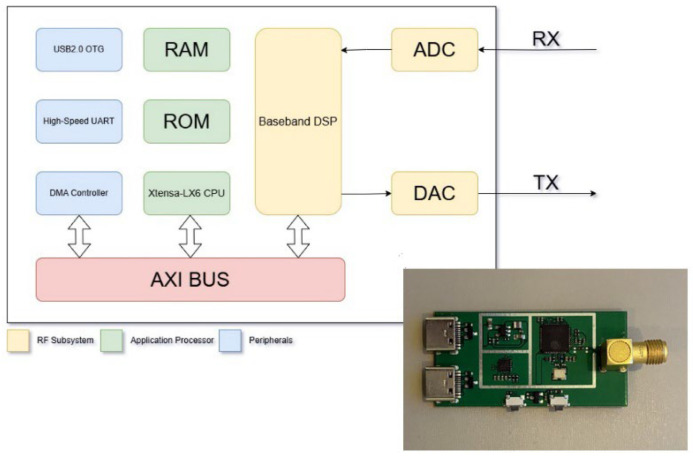
Internal architecture and physical appearance of the ESP32-S3 dongle. Arrows denote the data-flow paths between the RF subsystem, signal-conversion modules (ADC/DAC), baseband DSP, processor, memory, and peripheral units. The pink-colored AXI bus serves as the primary high-bandwidth communication backbone, facilitating efficient data transfer among all major on-chip components.

In this study, the ESP32-S3 Dongle (Figure 4) is employed as the CSI data acquisition platform. ESP32-S3 is a low-power, compact microcontroller (MCU) that integrates a Wi-Fi RF transceiver, is compatible with the IEEE 802.11n standard and supports both 20 MHz and 40 MHz bandwidth configurations. It offers an accessible C/C++ development environment alongside dedicated CSI data acquisition libraries.

**Figure 5:**
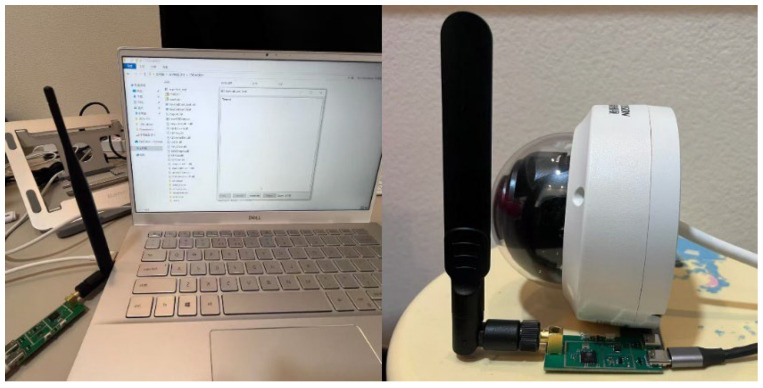
Connection diagram illustrating the setup of the transmitter, receiver, and a computer.

For data collection, two ESP32-S3 Dongles with different firmware configurations are utilized: one operating as a transmitter and the other operating as a receiver. Upon activation, the transmitter autonomously initiates the transmission of empty data packets. Concurrently, the receiver establishes a connection with the transmitter and enters an active state, receiving these packets and extracting the associated CSI feature information. The receiver then communicates with a PC via a serial port (Figure 5), transmitting the CSI feature data at a rate of 100 frames per second. A dedicated data capture application on the PC records the CSI data in a LevelDB database for subsequent processing.

**Figure 6:**
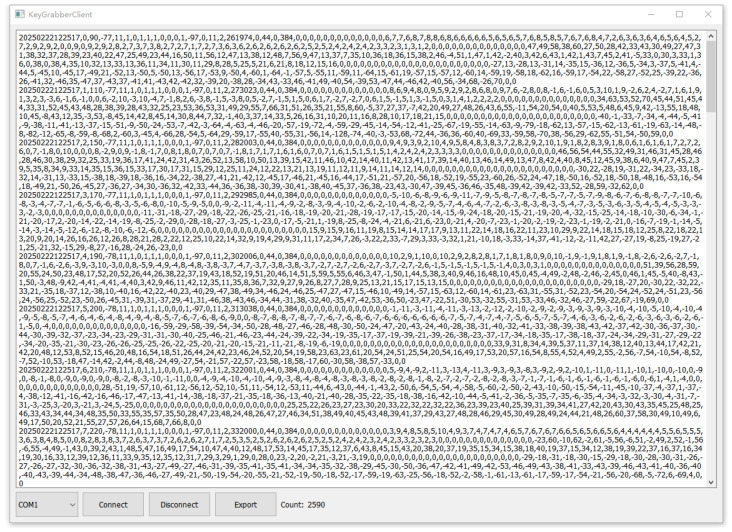
User interface of the data capture software displaying the real-time data logs being recorded.

The data capture software (Figure 6) systematically stores raw CSI data at a frequency of 100 entries per second. Each entry comprises a timestamp alongside auxiliary metadata such as signal strength and device MAC addresses. However, this study exclusively focuses on the CSI features themselves. These features are stored sequentially in subcarrier order, preserving both the real and imaginary coefficients for each CSI entry. Additionally, the capture software interface enables real-time monitoring of sampling values during the data acquisition process.

#### 4.1.2. Data Acquisition Environment

**Figure 7:**
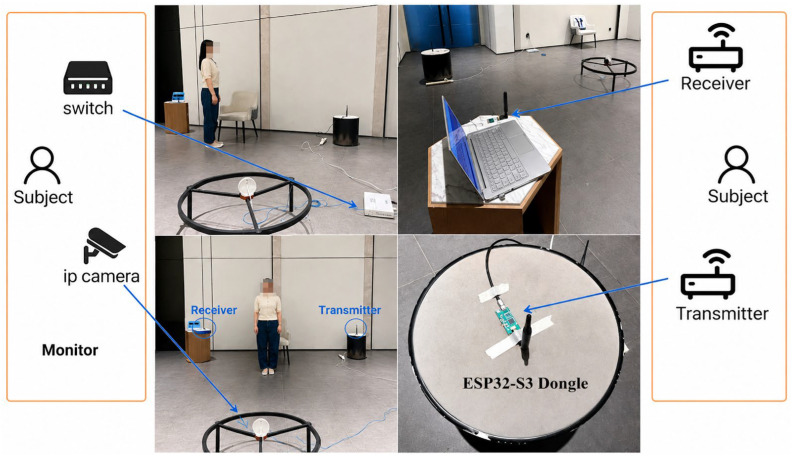
Live view of the data acquisition environment, showing the positioning of the transmitter, receiver, and volunteer performing actions.

A relatively empty room was selected for the experiment, where the transmitter and receiver were positioned at different locations. A camera was used to continuously record the scene, with timestamps on the video to help identify and differentiate various actions. The volunteers performed several distinct actions between the transmitter and receiver, as illustrated in Figure 7.

#### 4.1.3. Data Overview

The parameters of the receiver side of the collector are configured as follows:

.lltf_en = true,

.stbc_htltf_en = false, .stbc_htltf2_en = false,

.stbc_htltf2_en = false,

.ltf_merge_en = true, 

.channel_filter_en = false, 

.channel_filter_en = false, .manu_scale = false,

.shift = false,

In this experiment, the legacy long training field (LLTF) is activated, with the filter disabled. The channel bandwidth is set to 20 MHz, and each transmission of CSI data contains 52 subcarriers, each organized as real and imaginary components, resulting in 104 coefficients per data group. The data are transmitted at a frequency of 100 Hz, meaning that a new set of data is sent every 10 ms.

A volunteer performs seven different postures: lying down, falling, walking, picking up, running, sitting down, and standing up, along with a “no one” state, totaling 200,000 data groups. The entire dataset has a size of 104 × 200,000 × 200,000.

### 4.2. Data Pre-Processing

**Figure 8:**
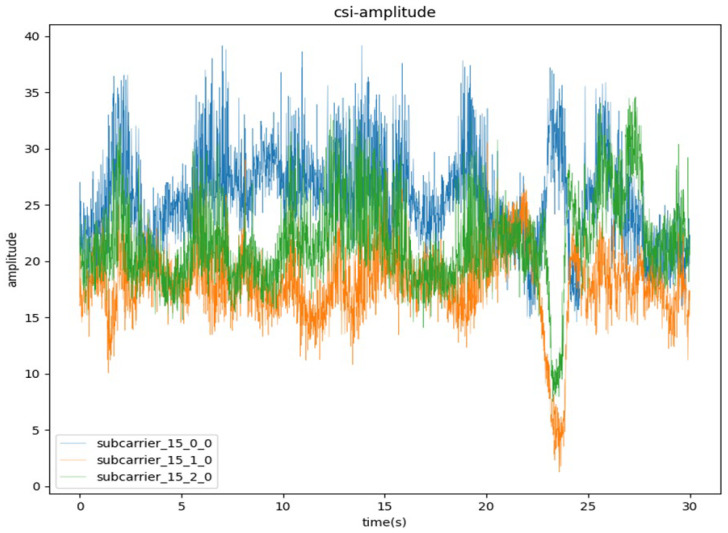
Amplitude of the CSI data before pre-processing.

**Figure 9:**
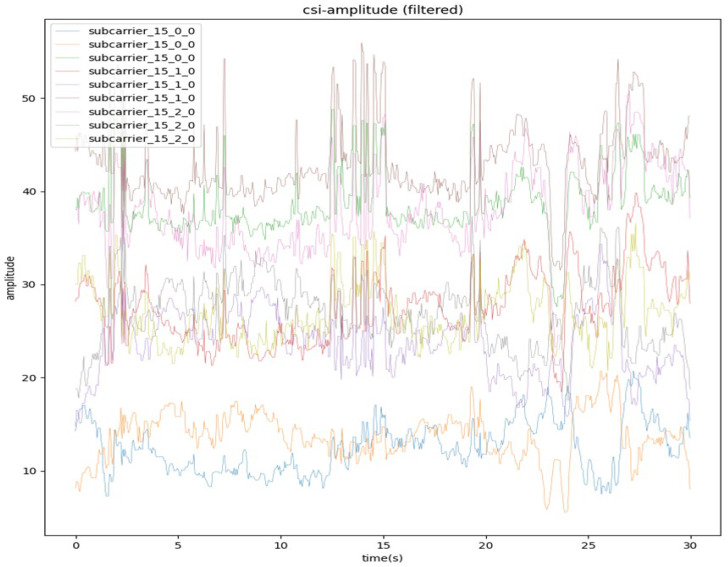
Amplitude of the CSI data after median and low-pass filtering.

**Figure 10:**
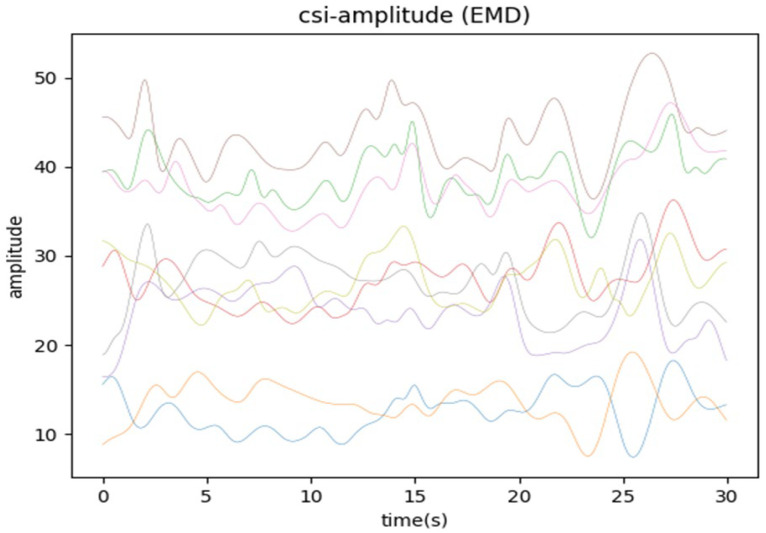
Amplitude of the CSI data after high-frequency component removal using EMD. The different coloured lines represent the reconstructed low-frequency CSI amplitude signals from multiple subcarriers after discarding the high-frequency IMFs. Each curve shows how a specific subcarrier’s amplitude evolves over time once noise-dominated components have been removed.

The pre-processing of the CSI data begins by calculating the amplitude values of the CSI data as shown in Figure 8. Afterward, both median filtering and low-pass filtering are applied to the amplitude data using an eighth-order Butterworth filter to remove noise and smooth the signal, as illustrated in Figure 9. Finally, EMD is employed to extract and remove high-frequency components from the data, enhancing the signal quality for further analysis, as depicted in Figure 10.

### 4.3. Model Construction

#### 4.3.1. Training Dataset Preparation

The pre-processed training set consists of 49 features, derived from 160,000 out of 200,000 sets of data. The data are grouped into 200 samples per batch to ensure that all actions within each group are consistent. As a result, the training set has dimensions of 49 × 200 × 80,049, corresponding to seven gesture categories and two states (manned and unmanned). All the data are labeled appropriately and undergo normalization. The remaining data are grouped similarly and used as the test set.

#### 4.3.2. Network Structure

**Figure 11:**
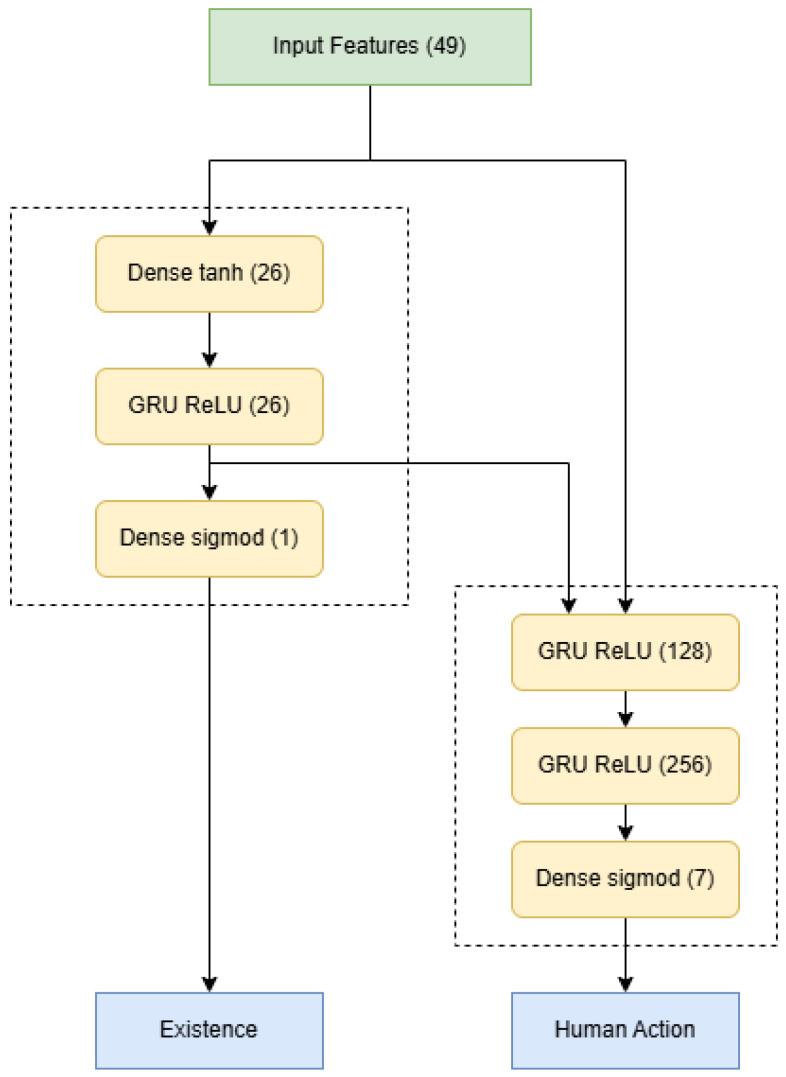
Network topology of STAR, featuring a multi-layer GRU architecture for action classification and person presence detection. The arrows show the forward flow of CSI features, the colours distinguish different layer types, the dashed boxes group the two output branches, and the numbers indicate the unit size of each layer.

As illustrated in Figure 11, the network uses a three-layer GRU architecture within an RNN. To enhance the system’s robustness, the detection of occupied and unoccupied states is incorporated. This helps distinguish noise signals from action signals during unoccupied states, reducing the risk of false alarms in action classification. By replacing LSTM with a GRU, the network structure is simplified, which lowers computational demands and facilitates easier deployment on edge devices. Cross-entropy is used as the loss function for both person presence detection and action classification.

#### 4.3.3. Model Training

The training framework is implemented using PyTorch 2.0.1. The batch size is set to 200, representing the group size, with 200 iterations in total. The model achieves an average classification accuracy of 93.52% across 80,000 test samples. The classification accuracy for each category is shown in Table 1. A per-class performance discussion follows—the fall class exhibits lower accuracy compared to other activities. This is expected because fall events are short, abrupt, and often resemble transitions into lying-down postures, making them difficult to distinguish in CSI amplitude space. Similar confusion patterns have been reported in prior CSI-based HAR studies.

#### 4.3.4. Impact of Pre-Processing Steps on Accuracy and Computational Cost

A detailed analysis of the pre-processing pipeline shows that each stage contributes differently to both recognition accuracy and computational overhead. Median filtering stabilizes the raw CSI amplitude by removing impulsive noise and measurement spikes, improving the reliability of static-activity classification while adding only about 0.12 ms of processing time per frame. The low-pass Butterworth filter provides the most substantial accuracy improvement by suppressing high-frequency fluctuations unrelated to human motion, thereby enhancing the separability of dynamic activities with a moderate computational cost of approximately 0.38 ms per frame. Empirical mode decomposition further refines the signal by isolating motion-related intrinsic mode functions, which is particularly beneficial for subtle or fine-grained activities, although this step introduces the highest overhead at roughly 1.95 ms per frame. When combined, these pre-processing operations increase overall HAR accuracy from 87.9% to 93.52% while keeping total latency below 2.5 ms per frame, ensuring that the system maintains real-time performance on the RV1126 platform.

### 4.4. Edge Computing

#### 4.4.1. Hardware Platform

Traditional CSI sensing systems typically rely on the computational resources of PCs or the cloud infrastructure, which introduce significant concerns regarding data privacy, deployment complexity, and associated costs. To address these challenges, we propose an offline deployment method that utilizes portable hardware devices for model inference, eliminating the dependency on PCs and cloud services. This approach enhances the practicality of Wi-Fi sensing technologies in real-world engineering applications.

Recent studies have explored the application of edge computing to Wi-Fi sensing, with implementations using MCU- or CPU-based platforms such as Raspberry Pi and ESP32, as well as GPU-based solutions such as the Jetson series of single-board computers. However, we argue that the computational power of the former is insufficient to ensure real-time data processing while maintaining inference accuracy. In contrast, while the latter provides substantial computational power, its large size and high power consumption hinder its deployment in field applications. Furthermore, both the Raspberry Pi and Jetson devices lack integrated CSI data capture modules, necessitating the use of external receivers, which further complicates deployment.

**Figure 12:**
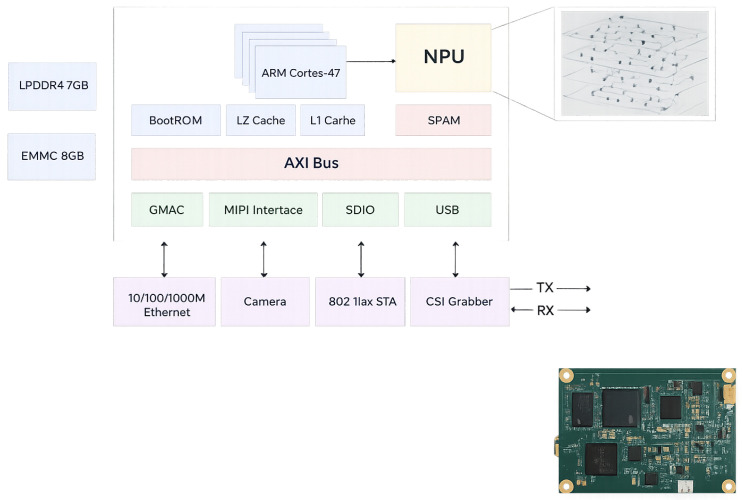
RV1126-based edge hardware. The arrows indicate data flow between on-chip modules, the coloured blocks highlight major functional units such as the CPU, NPU, memory, and I/O interfaces, and the dashed outlines group related subsystems within the SoC. The numbers label key components and memory capacities, while the board image below shows the corresponding physical hardware implementation.

In our experiments, we utilize a custom-built hardware platform based on the Rockchip RV1126 processor, which integrates the ESP32-S3, allowing for seamless CSI data acquisition and processing within a single device. A key feature of the RV1126 is its internal NPU, which is capable of 2 TOPS (tera operations per second), enabling real-time inference without overburdening the CPU (Figure 12). This integration significantly improves the efficiency and convenience of deploying Wi-Fi sensing technology in practical applications.

#### 4.4.2. Performance Evaluation

The proposed network comprises 97,616 weight parameters, with the core pre-processing and RNN inference computations implemented in vectorized C. The performance evaluation of our network is summarized in Table 2.

In CPU mode, the performance is assessed under both the FP16 and INT8 quantization formats, using a 1.5 GHz Cortex-A7 processor integrated within the RV1126 platform. When switching to the NPU mode, the model undergoes INT8 quantization via Rockchip’s toolchains, resulting in a test inference speed of 33 MHz. This represents a six-fold increase in performance compared to that of the CPU mode. Furthermore, in the NPU mode, the CPU is solely engaged in data pre-processing, reducing its occupancy to 8%. Notably, the use of INT8 quantization does not significantly impact the inference accuracy, thereby balancing enhanced computational efficiency with minimal loss in performance.

#### 4.4.3. Real-World Applicability in Dynamic Environments

To further evaluate the robustness of STAR in practical deployment scenarios, additional experiments were conducted in dynamic indoor environments with varying room layouts, furniture arrangements, and user behaviors. These tests were performed across multiple rooms with different sizes and multipath characteristics, including open living spaces, narrow corridors, and furnished office-style environments. Despite the increased variability in channel conditions, the system maintained stable performance, with overall recognition accuracy fluctuating within ±3.1% of the baseline. STAR also demonstrated resilience to user-dependent factors such as differences in walking speed, limb length, motion intensity, and individual movement styles. Notably, the system did not require user-specific calibration or environment-specific retraining, and the pre-processing pipeline effectively mitigated the impact of environmental noise and layout changes. These observations indicate that STAR generalizes well to heterogeneous indoor settings and diverse user behaviors, supporting its applicability in real-world smart-home, healthcare, and mobile IoT deployments where environmental conditions and human activity patterns are inherently dynamic.

#### 4.4.4. Latency Across Activity Types and System Load

To provide a more comprehensive understanding of STAR’s real-time performance, we evaluated end-to-end latency across all seven activity classes and under different system load conditions on the RV1126 platform. The measured latency includes CSI acquisition, pre-processing, NPU inference, and post-processing. Across all activities, the system maintained sub-second responsiveness, with average latencies ranging from 312 ms for short, high-energy motions (e.g., waving) to 427 ms for longer-duration activities (e.g., walking), where additional CSI frames are naturally accumulated before classification. These variations reflect differences in activity temporal structure rather than computational bottlenecks. Under increased system load—introduced by concurrent background processes such as data logging and auxiliary sensor polling—the latency remained stable, increasing by no more than 8.7%, owing to the isolation of inference on the dedicated NPU and the use of lock-free queues for data transfer. These results confirm that STAR sustains real-time performance across diverse activity types and operating conditions, reinforcing its suitability for latency-sensitive smart-home and mobile IoT applications.

#### 4.4.5. Scalability Across Heterogeneous IoT Hardware

Although the Rockchip RV1126 serves as the primary deployment platform in this study, STAR is designed to be portable across a wide range of IoT devices with varying computational capabilities. Following the scalability considerations discussed in recent ISAC literature—particularly the device-adaptation and hardware-heterogeneity analysis outlined in Localization in ISAC: A Review [42]—we evaluated how STAR’s pre-processing pipeline and GRU-based inference model would perform on devices with different CPU, memory, and accelerator configurations. On lower-end microcontroller-class platforms such as the ESP32-S3, the full pipeline cannot be executed in real time due to limited RAM and the absence of hardware acceleration; however, the pre-processing stages remain lightweight enough to run at 40–60 Hz, enabling hybrid deployments where inference is offloaded to a nearby edge gateway, consistent with prior MCU-based sensing frameworks [43,44]. Mid-range devices such as the Raspberry Pi 4B can execute the complete pipeline at 8–12 Hz using CPU-only inference, and performance increases to 20–25 Hz when using TensorFlow Lite with NEON optimizations, aligning with observations in embedded deep learning benchmarks [45]. Higher-end NPUs such as the RK3588 or Amlogic A311D can exceed 60 Hz throughput with INT8 quantization, demonstrating that STAR scales favorably with available compute resources, similar to performance trends reported in recent edge AI acceleration studies [46]. These observations indicate that the proposed framework is not tied to a single hardware platform and can be adapted to heterogeneous IoT environments by adjusting model size, quantization level, and deployment topology. This flexibility supports practical deployment across diverse smart-home, healthcare, and mobile sensing scenarios where device capabilities vary widely.

## 5. Conclusions

This work presented STAR, a Wi-Fi CSI-based human activity recognition framework built around a lightweight three-layer GRU architecture. The model achieves high classification accuracy with low computational overhead, making it suitable for deployment on resource-constrained edge devices. To support real-time operation, core computations were implemented using vectorized C code, enabling the RV1126 platform to sustain CSI sampling rates above 100 Hz and ensuring stable end-to-end performance in practical environments.

NPU acceleration further improves inference speed, providing up to a six-fold increase over CPU execution while reducing CPU utilization to approximately 8%. This allows the CPU to focus on data acquisition and pre-processing. Importantly, INT8 quantization preserves model accuracy, demonstrating that efficient deployment is feasible without degrading recognition performance.

By combining lightweight temporal modeling, hardware-aligned optimization, and an efficient signal processing pipeline, STAR offers a practical solution for real-time single-receiver Wi-Fi sensing. The system operates entirely on-device, providing a privacy-preserving alternative to cloud-based HAR and supporting scalable deployment in real-world settings.

STAR demonstrates the viability of low-power, real-time CSI sensing for edge AI applications. The framework lays the groundwork for future extensions in smart home monitoring, healthcare, and security systems, offering an energy-efficient and privacy-conscious platform for activity recognition.

## Figures and Tables

**Table 1:** Model classification accuracy.

Class of Activity	Class of Activity
Lie down	96.12%
Fall	85.22%
Walk	90.11%
Pickup	94.55%
Run	88.71%
Sit down	96.90%
Stand up	97.46%
Human presence or absence	99.11%

**Table 2:** Performance evaluation in FP16 and INT8 quantization mode.

Quantization Accuracy	Required Arithmetic Power	Reasoning Speed	CPU Occupancy
INT8	48 Mflops	5000 KHz	28%
FP16	166 Mflops	1800 KHz	56%

## Data Availability

All datasets used in this study were collected by the authors in controlled laboratory environments using custom-built hardware and acquisition systems. The data are not publicly available due to privacy and institutional restrictions but can be made available from the corresponding author upon reasonable request.
